# Plant Immunity and Crop Yield: A Sustainable Approach in Agri-Food Systems

**DOI:** 10.3390/vaccines9020121

**Published:** 2021-02-03

**Authors:** Marcello Iriti, Sara Vitalini

**Affiliations:** 1 Department of Agricultural and Environmental Sciences, Università degli Studi di Milano, 20122 Milano, Italy; sara.vitalini@unimi.it; 2 National Interuniveristy Consortium of Materials Science and Technology (INSTM), 50121 Firenze, Italy; 3 BAT Center—Interuniversity Center for Studies on Bioinspired Agro-Environmental Technology, University of Napoli “Federico II”, 80055 Napoli, Italy

**Keywords:** innate immunity, systemic acquired resistance, plant activators, elicitors, priming

## Abstract

Innate immunity represents a trait common to animals and plants. Indeed, similar to animals, plants also evolved a complex defense machinery to defend against pest and pathogen attacks. Due to the concerns posed by the intensive use of agrochemicals, the possibility to stimulate the plant immune system with environmentally friendly and low-risk chemical and biological inducers is intriguing. Therefore, some plant protection products are commercially available to trigger the plant’s immune system, with benefits in terms of consumer health and environmental protection.

In their natural ecosystems, plants evolved a complex defense system to protect themselves from pathogen and parasite attacks. Indeed, many harmful (micro)organisms can damage plants, including arthropods, nematodes, phanerogamic parasites, fungi and oomycetes, bacteria and phytoplasmas, as well as the simplest infectious agents, viruses and viroids. In addition, plants are sessile organisms which cannot escape the harsh environment due to adverse meteorological conditions, extreme temperatures, high solar irradiance, UV radiation and exposure to both natural and anthropogenic pollutants. Therefore, the evolutionary success and survival of plant species has depended on their ability to counteract biotic and abiotic stressors and cope with challenging environmental conditions. However, the natural course of evolution has been altered by man since the beginning of agriculture (around 10.000 years ago). Indeed, in the agroecosystems, cultivated plants are more susceptible to infections, due to intensive monoculture, breeding and varietal selection, according to the paradigm” to produce or to defend”, resulting in the (massive) use of agrochemicals in crop protection and the associated risks and concerns [1].

Plants can distinguish among the self, non-self and altered self. Their immune system consists of two perception systems able to recognize pathogens: pattern-triggered immunity (PTI) and effector-triggered immunity (ETI). Highly conserved pathogen-associated molecular patterns (PAMPs), microbe-associated molecular patterns (MAMPs) and herbivore-associated molecular patterns (HAMPs) are recognized by membrane pattern recognition receptors (PRRs), as well as endogenous damage-associated molecular patterns (DAMPs) released by the damaged host tissues, thus triggering PTI. Therefore, taxonomic groups of parasites featuring a specific pattern (e.g., fungal chitin or bacterial flagellin) can be sensed by a PRR, the latter including receptor-like kinases and receptor-like proteins. ETI is activated by effector proteins encoded by pathogen avirulence (*avr*) genes and secreted into plant cells, where they are recognized by intracellular nucleotide-binding domain leucine-rich repeat (NLR)-type receptors encoded by resistance (*R*) genes. Intriguingly, epigenetic mechanisms significantly affect the plant immune response. Indeed, defense-related gene expression involves epigenetic modifications (histone acetylation and methylation, DNA methylation) that contribute to immunogenic memory. Epigenetic priming does not require any change in the DNA sequences, affecting the dynamical chromatin states, and exerts a transgenerational effect, i.e., priming states are heritable [2].

In this scenario, any strategy aimed at minimizing the chemical control of plant diseases is mandatory, including the possibility of stimulating the plant’s immune system with chemical (synthetic and natural) and biological (bacteria, mild viruses, and weak strains) inducers [1]. The plant defense armamentarium consists of preformed (or passive) and inducible (or active) mechanisms, each in turn divided into chemical and structural defenses. These typically include, for example, among active defenses, oxidative burst and programmed cell death (reminiscent of animal respiratory burst and apoptosis), cell wall strengthening, expression of pathogenesis-related (PR) proteins and increased levels of antimicrobial secondary metabolites (phytoalexins), as recently emphasized. Indeed, seaweed extracts rich in β-glucan elicited a broad defense response in tomato plants infected with the fungus *Fusarium oxysporum*, consisting of increased enzyme activity of peroxidase (involved with H_2_O_2_ in cell wall lignification), β-1,3-glucanase (a PR protein) and phenylalanine ammonia lyase (the key enzyme of the biosynthesis of phenolic phytoalexins) [3]. The role of modifications of cell wall structure and composition as an immune response was highlighted in transgenic tobacco plants overexpressing polygalacturonase-inhibiting protein (directed toward the lytic enzymes of pathogens and involved in the release of oligogalacturonides, i.e., DAMP—damage-associated molecular patterns) and cinnamyl alcohol dehydrogenase (responsible for the biosynthesis of monolignols, the building blocks of lignin) [4]. Beneficial bacteria *Bacillus subtilis* and *Pseudomonas fluorescens* triggered immunity in *Arabidopsis thaliana* against *Botrytis cinerea* and *Pseudomonas syringae*, orchestrating the phytohormone levels and increasing the gene expression of PR1, PR4 and defensins [5]. PR proteins can also be involved in crosstalk between proteins through the formation of multiprotein complexes, thus amplifying the host defense response. This is the case in GmPR8 interacting with serine hydroxymethyltransferase and soluble NSF (N-ethylmaleimide sensitive fusion) attachment protein in soybean plants resistant to cyst nematodes [6]. Accelerated molecular breeding programs for pest resistance should also be considered, such as the genome-wide association mapping approach, successfully applied in rice to develop new cultivars resistant to brown planthopper, one of the most important pests of this crop [7].

In conclusion, crop yield and food security strictly depend on plant health (and immunity), a crucial link in the current and future global demographic and climatic scenario. Therefore, to feed the planet represents the major challenge of modern agriculture, to be faced sustainably. This implies the (low) use of environmentally friendly plant protection products, including elicitors and plant activators [8]. It is noteworthy that plant “vaccination” does not pose any risk of selecting pathogen strains resistant to agrochemicals, but on the contrary, triggers the host multigenic defense machinery. This represents a very relevant issue due to the global public health threat of antimicrobial resistance. In addition, induced immunity may protect plants from incurable diseases caused by bacteria, including phytoplasmas and viruses (of course, conventional antibiotics and antivirals cannot be used in agriculture). Not least, plant activators and elicitors are, in general, less toxic than agrochemicals with biocidal mechanism, though they present a major drawback due to the limited number of registered ”vaccines” available on the market [8]: in brief, more studies are needed in this field, i.e., increased funding from government bodies and multinational pharma companies.

## Data Availability

Not applicable.
